# Effect of Chest Physiotherapy Technique on Bilateral Bronchial Pneumonia Secondary to Acute Respiratory Distress Syndrome: A Case Report

**DOI:** 10.7759/cureus.50437

**Published:** 2023-12-13

**Authors:** Urvini R Lokhande, Vaishnavi M Thakre, H V Sharath

**Affiliations:** 1 Department of Paediatric Physiotherapy, Ravi Nair Physiotherapy College, Datta Meghe Institute of Higher Education and Research, Wardha, IND

**Keywords:** rehabilitation, physical therapy, physiotherapy, bronchial pneumonia, acute respiratory distress syndrome

## Abstract

This case report investigates the impact of a specific chest physiotherapy technique on a patient with bilateral bronchial pneumonia secondary to acute respiratory distress syndrome (ARDS). ARDS is a life-threatening condition characterized by severe respiratory failure, and bronchial pneumonia can further complicate the clinical course. The chosen chest physiotherapy technique aims to improve respiratory function and alleviate symptoms in the context of this challenging scenario. ARDS can develop in individuals who are seriously injured or have other severe conditions. ARDS is characterized by insensitive cyanosis, declining lung compliance, and high morbidity in intensive care units. It is a complicated and accumulating condition that develops from acute damage to the lungs. The case involves a detailed examination of a patient diagnosed with bilateral bronchial pneumonia as a complication of ARDS. The application of a targeted chest physiotherapy technique is described, emphasizing its methodology and the rationale behind its selection. Through this case report, we aim to contribute valuable insights into the potential efficacy of the specific chest physiotherapy technique for managing respiratory complications associated with ARDS-induced bilateral bronchial pneumonia. The findings may have implications for clinical practice, guiding healthcare professionals in tailoring interventions for similar cases and optimizing patient care in critical respiratory conditions. Additionally, the report underscores the importance of individualized approaches in the management of complex respiratory disorders, highlighting the need for further research to validate and refine such therapeutic strategies. The report delves into the patient's response to the intervention, documenting any observable improvements in respiratory parameters, lung function, and overall clinical outcomes. There were numerous etiologists, and it frequently ended in intense respiratory failure; after that death, the majority of care is supportive and concentrates on treating the underlying cause as well as providing ventilation. Physical therapy should begin as soon as the ARDS is treated. In this case, we discuss and conclude the various aspects of physiotherapy interventions for bilateral bronchial pneumonia secondary to ARDS. Chest physiotherapy plays an important role in respiratory conditions for breathing effectiveness and to reduce airway resistance.

## Introduction

Acute respiratory distress syndrome (ARDS) has characteristics of non-cardiogenic pulmonary oedema, extreme hypoxemia, and bilateral chest radiographic opacities [1]. It is characterized by insensitive cyanosis, declining lung compliance, and high morbidity in intensive care units (ICU). The main causes of pulmonary ARDS are pneumonia or trauma without additional associated infections. Extrapulmonary ARDS is defined as having no obvious symptoms of pneumonia or pulmonary infection and being brought on by a central nervous system infection, an intra-abdominal bloodstream infection, or a catheter-related bloodstream infection [2].

ARDS is a complicated and accumulating condition that develops from acute damage to the lungs. There were numerous etiologists, and it frequently ended in intense respiratory failure after that death [3]. The majority of care is supportive and concentrates on treating the underlying cause as well as providing ventilation. Ashbaugh et al. coined the word ARDS in 1967 to describe a disease characterized as "an acute onset of rapid and shallow breathing, oxygen deprivation, and decreased compliance after a variety of stimuli" [4]. Acute eosinophilic pneumonia, immune-mediated pulmonary hemorrhage and vasculitis, infection, collagen vascular conditions, drug side effects, injectables, inhalants, shock, and radiation pneumonitis are just a few of the numerous causes of ARDS [5]. Sepsis, a severe and pervasive bloodstream infection, inhaling hazardous substances, and other factors are the most frequent causes of ARDS. Pneumonia is often the final phase of an infectious disease in older adults, including the 2019 coronavirus disease, chronic alcoholism, and long-term tobacco chewing [6].

Elderly people are more susceptible to pneumonia because of their weak gag reflex [7]. Pathogens that are not visible by gram staining and cannot be produced via conventional techniques are the cause of atypical pneumonia. *Legionella *species, *Chlamydia pneumoniae*, and* Mycoplasma pneumoniae* are the most frequently causing organisms of atypical pneumonia. Atypical pneumonias require a different therapeutic strategy than typical pneumonias [8]. Patients with severe forms of ARDS who require ventilatory support exhibit weakness of muscle and reduced exercise tolerance; as a result, immediate treatment therapy begins during the initial phase of the disease stage. Patients with ARDS may benefit from early physical care as a transitional step towards rehabilitation. ARDS is a condition marked by dyspnea, fatigue, chest pain, and intellectual disturbances. These symptoms can lead to reduced capacity for exercise, functional decline, and, ultimately, a poor quality of life [9]. Treatments that maintain the airway, ensure a sufficient amount of oxygen, and help to maintain function are necessary for treating patients with ARDS. Adequate perfusion, proper positioning, protocol weaning the patient, protective lung ventilation, and avoiding further complications are all parts of supportive therapy that are the 5 Ps [10].

The most significant factor triggering ARDS is lung vascular injury. The growth of protein-rich pulmonary emboli is strongly supported by evidence that even when lung vascular pressure is within normal limits, the lung endothelium may be damaged in a number of ways, but the neutrophil-dependent damage to the lung's pathway may be the one that has received the most research. Neutrophils are the main factor in lung damage [11]. Prone positioning can improve pao2 and lower paco2 [12]. The most prevalent disease affecting people of all ages worldwide is pneumonia; the mainstay of treatment for pneumonia is antibiotics, with supportive therapies implementing the majority of other therapies. Despite the lack of solid evidence, chest physical therapy has been extensively used as adjuvant therapy for adult pneumonia [13]. Hence, physiotherapy is a must for improving breathing and reducing airway resistance.

## Case presentation

We present a case of a 48-year-old male patient who arrived at the hospital with complaints of a rise in temperature for five days and coughing with expectorants for four days, which was yellowish-green in colour, purulent, and thick sputum. Laboratory analysis reveals a low white blood cell (WBC) and platelet count; budding yeasts are found in urine cultures; and an X-ray was performed that suggested bronchial pneumonia. He has been advised to shift to the medical intensive care unit (MICU) and was referred to physiotherapy for further treatment.

The patient was tachypneic. He was conscious and well-oriented to time, place, and person. On observation, the patient has chest asymmetry with restricted movement of the chest on both sides, along with the use of accessory muscles. Bilateral crepitus was present on auscultation in the upper zone, and air entry was decreased. No other medical history was discovered. He was taking drugs like Dolo 650 mg (Micro Labs, Bengaluru, India), Mucinac 600 mg (Cipla, Mumbai, India), and Tamiflu 75 mg (Roche Diagnostics, Chennai, India) for the present complaints. The pre-treatment outcome measures are given in Table 1.

**Table 1:** Pre-treatment outcome measures NPRS: Numerical Pain Rating Scale; MMRC: Modified Medical Research Council; WHO-QOL: World Health Organization Quality of Life

Serial number	Scales which were used in patient	Score
1	NPRS	8 out of 10
2	MMRC	Grade 4
3	WHO-QOL	58 out of 100
4	Emotional interaction	80 out of 100
5	Social interaction	82 out of 100
6	Environmental performance	66 out of 100

Investigatory findings

The chest X-ray was done in posteroanterior view, which shows hyper-translucency of the lung field with prominent bronchovesicular markings and hilar markings.

Hyper-translucency of the Lung Field

ARDS often leads to diffuse damage to the lung tissue, resulting in increased air spaces and reduced lung density, which can manifest as hyper-translucency on the X-ray.

Prominent Bronchovesicular Markings

In ARDS, the lung parenchyma may exhibit changes that affect the bronchial structures. The prominent bronchovesicular markings may be indicative of inflammation, fluid accumulation, or changes in the airway associated with the ARDS process.

Hilar Markings

**Figure 1:**
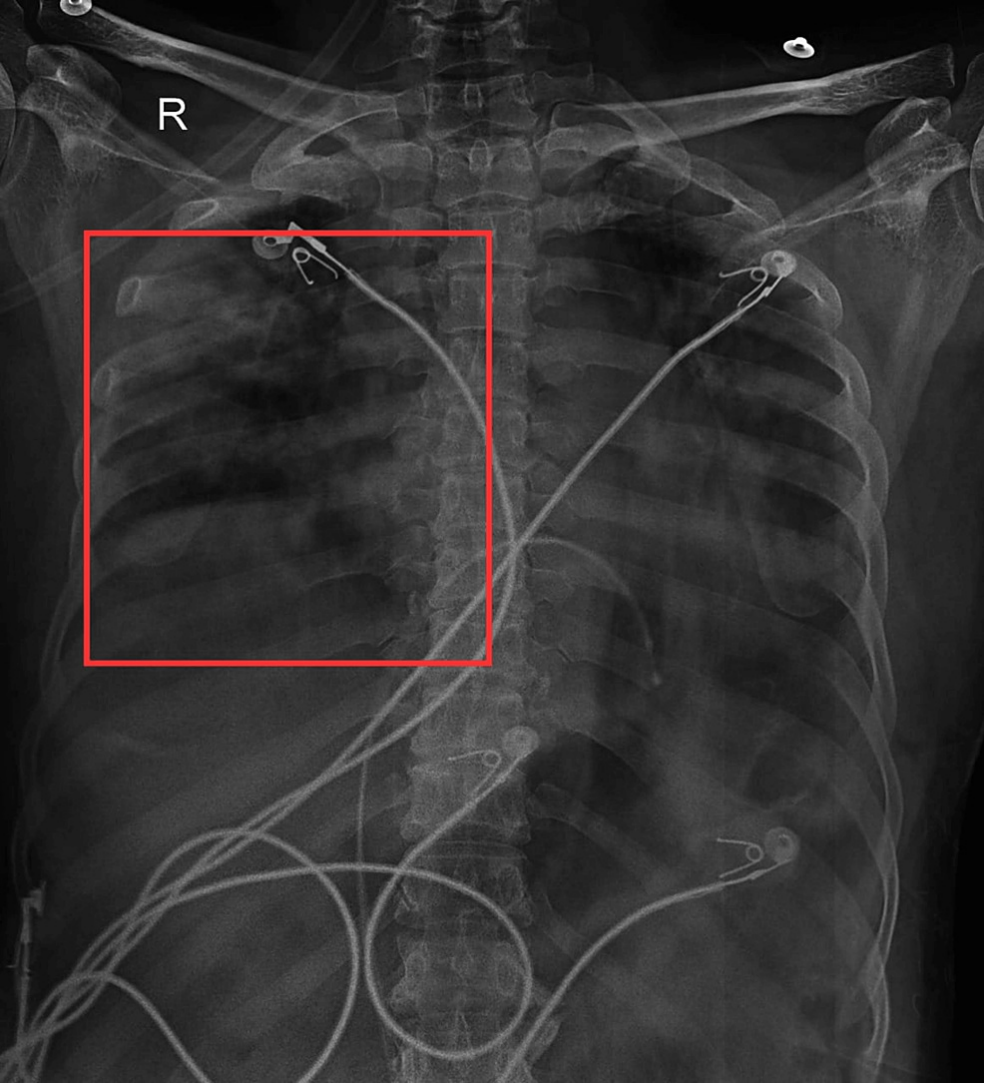
Chest X-ray in posteroanterior view Square represents hyper-translucency of the lung field with bronchovesicular markings and hilar markings present

The hilar markings can be influenced by factors such as vascular changes, lymphadenopathy, or inflammatory processes in ARDS. The X-ray may reveal increased prominence or changes in the hilar region (Figure 1).

Therapeutic interventions

**Figure 2:**
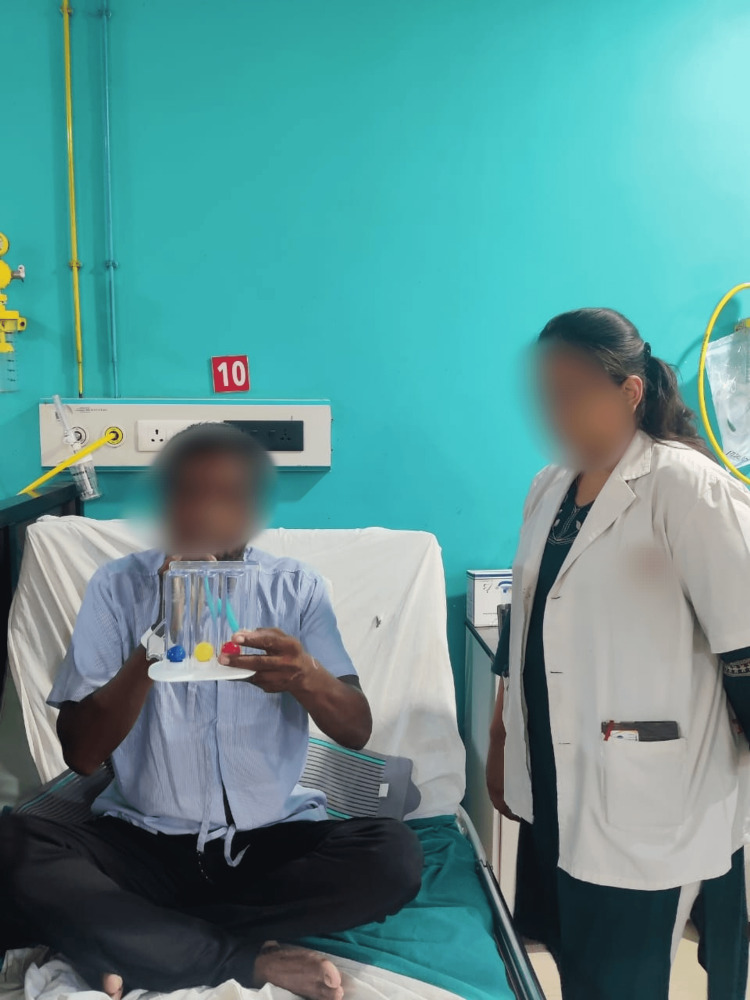
Patient undergoing incentive spirometry technique to enhance lung capacity

**Figure 3:**
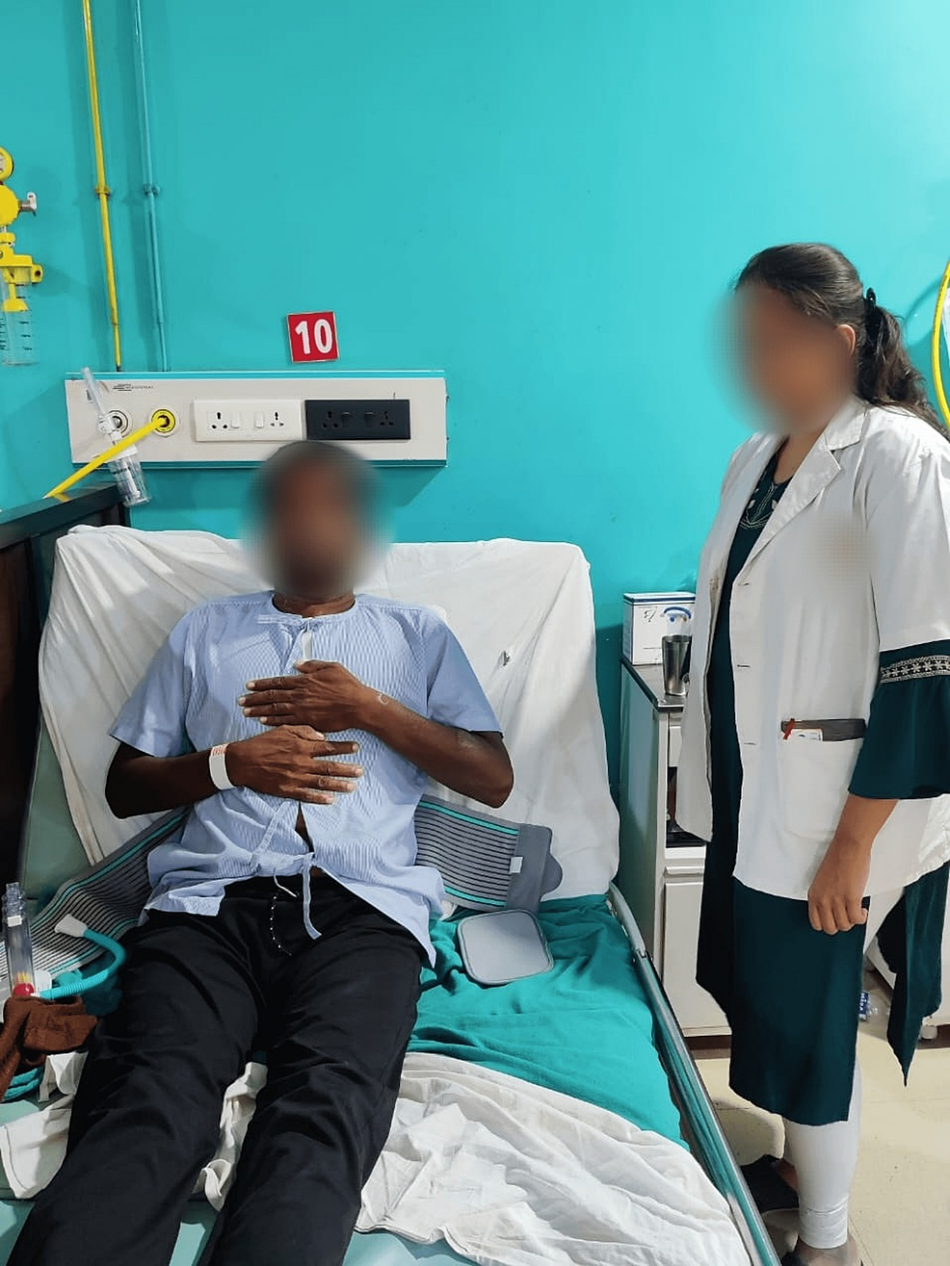
Patient undergoing diaphragmatic breathing exercise for relieving dyspnea

**Figure 4:**
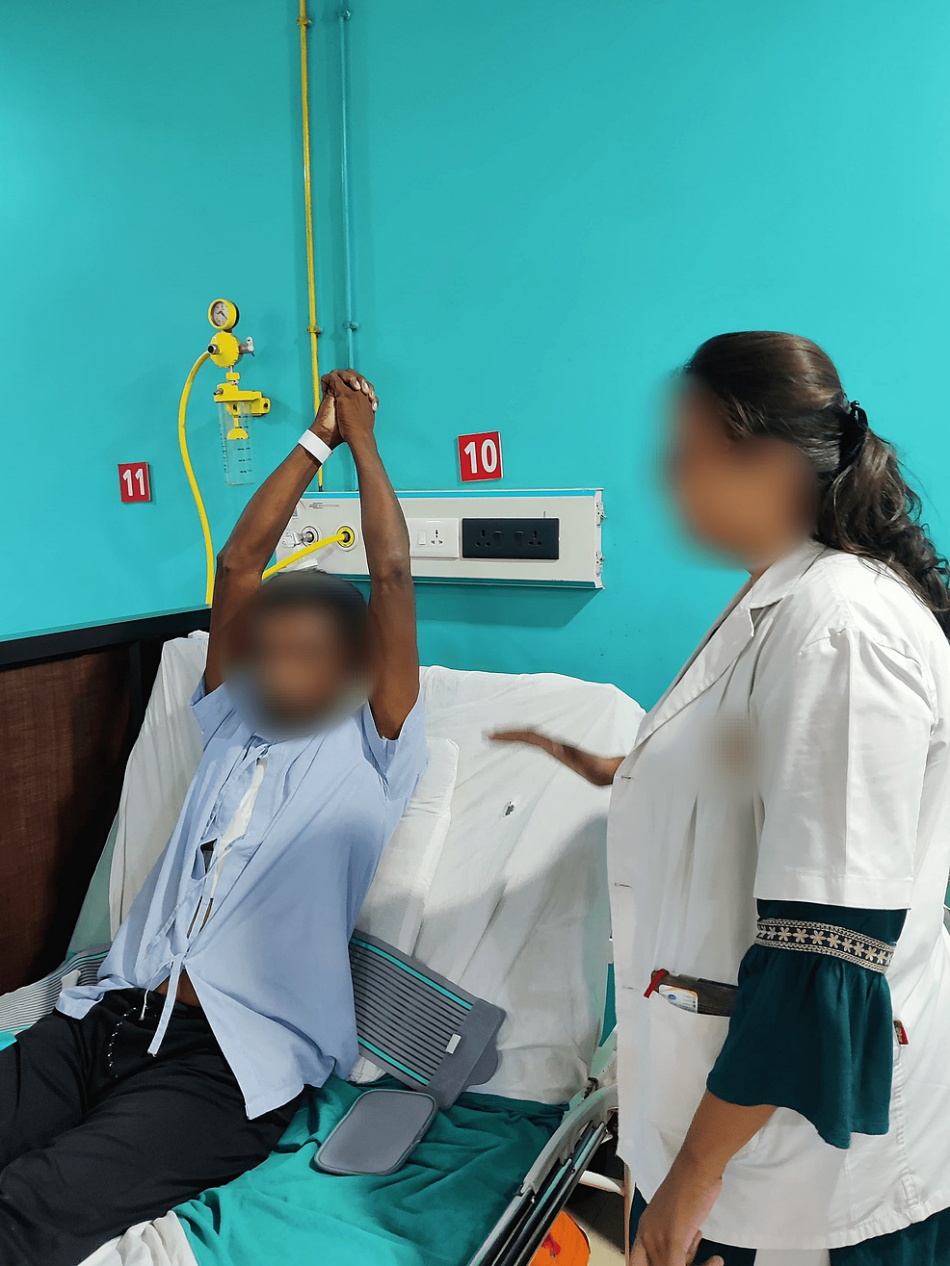
Patient undergoing thoracic expansion exercise

After informing the patient about his condition, permission was obtained to proceed with the procedure, which went as planned. The patient was treated with a multidisciplinary approach that was used to improve his recovery by increasing air entry. In order to improve breathing effectiveness and reduce airway resistance, we first work on removing the secretions to aid in improving the airway. To improve air passage, aerosol treatment and nebulization were administered three times per day for a week. The treatment was successful based on the outcome measures that were obtained during the assessment, and accordingly, interventions were given to the patient (Table 2 and Figure 2, Figure3, and Figure 4).

**Table 2:** Physiotherapy interventions Reps: repetition; cc: cubic centimeter; ROM: range of motion; NA: not applicable

Serial number	Intervention objectives	Clinical intervention	Frequency
1	Patient education	The treatment protocol was explained to the patient	NA
2	To improve respiratory rate and breathing pattern	Diaphragmatic breathing exercise	10 reps×1 set
3	To enhance the lung capacity through spirometry	Utilizing flow-oriented incentive spirometry	Three-second hold on 600 cc with 10 reps
4	To remove the secretions	Postural drainage: hands-on techniques such as percussion and vibration	Twice a day
5	To improve lung function and lung capacity	Breathing control, thoracic Expansion exercise, and forced expiration	Twice a day
6	To maintain the mobility of the patient	Active ROM exercise for both upper and lower extremities	First, start with 10 reps once a day and afterwards 10 reps in two sets three times per day for four weeks

Follow-up and outcome measures

The patient was able to carry out daily tasks with assistance after a week of rehabilitation, and symptoms like breathlessness were reduced. He changed his perspective on life to one of optimism. For four weeks, an exercise program with two to three sessions per day was conducted in the hospital. After that, the patient's ability to tolerate exercise improved. The patient could move around without difficulty, and his dyspnea grading converted from 4 to 2. The patient can then perform an incentive spirometry test with 900 cubic centimeter (cc) with a 10-second hold. Post treatment, the saturation level of 95% on room air is maintained. All post-treatment outcome measure scores are listed in Table 3.

**Table 3:** Post-treatment outcome measures NPRS: Numerical Pain Rating Scale; MMRC: Modified Medical Research Council; WHO-QOL: World Health Organization Quality of Life

Serial number	Scales which were used in patient	Score
1	NPRS	4 out of 10
2	MMRC	Grade 2
3	WHO-QOL	89 out of 100
4	Emotional interaction	88 out of 100
5	Social interaction	90 out of 100
6	Environmental performance	75 out of 100

## Discussion

The main causes of pulmonary ARDS are pneumonia or trauma without additional associated infections. Due to extensive airway collapse, which leads to oxygen deprivation and reduced ventilatory perfusion, oxygenation rather than ventilation is the primary problem with ARDS [14]. The lung's capacity is significantly lower than normal, and there are significant variations in alveolar compliance and time constant [15]. According to Masclans et al., a pulmonary rehabilitation program sought to target and address both the patient's immediate problems and any future challenges that might arise. The main facets of physiotherapy management were pulmonary rehabilitation and counselling. The rehabilitation techniques aimed to improve quality of life by decreasing cough rate, improving ventilation, and minimizing sleep disruption [16].

The patient's lack of literacy and the family's disengagement posed the only difficulties in managing this case, beginning with the administration of patients to the ICU on ventilatory support. This has been demonstrated in some research using an ICU mobility team and a quasi-experimental design. This early health program was significantly associated with shorter hospital stays, even though it was unrelated to a rise in subsequent issues [17].

Previous research has shown that physical function limitations in people with ARDS can be harmful in the short and long term. To deal with these problems, a pulmonary rehabilitation trial that combines modified strength training, aerobic training, and breathing exercises was developed. Pulmonary rehabilitation has been shown in numerous studies to enhance the capacity of exercise, sleep quality, depression, and general health-related quality of life. When treating serious ARDS with constant hypoxemia, particular care must be followed. Some studies using pulmonary rehabilitation have shown full functional recovery in patients with ARDS [18].

A placebo-controlled trial of physiotherapy in patients who were diagnosed with primary pneumonia was conducted. Randomly selected patients received postural drainage, external breathing assistance, percussion, vibration, and counselling on expectoration, deep breathing, and avoiding thrombosis through activity. In both groups, the same pharmacological management principles were applied. There was indisputable proof that regular physical treatment was beneficial throughout the illness's acute stage. Conversely, physical therapy seemed to lengthen hospital stays and the duration of fever in younger individuals, smokers, and those with interstitial pneumonia [19]. A new technique used to enhance gas exchange in patients suffering from ARDS involves shifting the patient from a supine lying position to a prone lying position [20].

Several outcomes were used for the assessment of how well the patient's therapy had worked, which included the Numerical Pain Rating Scale (NPRS), Modified Medical Research Council (MMRC), and World Health Organization Quality of Life (WHO-QOL). All the scales and questionnaires were reviewed on the first day of the physical therapy examination, on the day of discharge, and on the day of follow-up calls. Each metric had significantly improved, as evidenced by the variation in scores over these three days. By the use of incentive spirometry, segmental breathing, chest expansion exercises, and diaphragmatic exercises, lung compliance and breathing quality are improved.

## Conclusions

Patients with severe ARDS who need mechanical ventilatory support are most likely to have weak respiratory muscles and be less tolerant of physical exertion. For this reason, initial rehabilitation treatment that begins during the initial phase is crucial for enhancing their physical function. Physical therapy should begin as soon as the ARDS is treated. However, after undergoing our meticulously planned pulmonary rehabilitation program, the patient's cough intensity, breathlessness, lung function, weakness, and general quality of life all markedly improved. It is appropriate to assume that our all-encompassing strategy will lead to a change in the patient's respiratory condition.
